# TSH inhibits SERCA2a and the PKA/PLN pathway in rat cardiomyocytes

**DOI:** 10.18632/oncotarget.9393

**Published:** 2016-05-16

**Authors:** Jiajia Dong, Cuixia Gao, Jing Liu, Yunshan Cao, Limin Tian

**Affiliations:** ^1^ Department of Endocrinology, Gansu Provincial Hospital, Lanzhou, Gansu, China; ^2^ Department of Ultrasonic Diagnosis, Gansu Provincial Hospital, Lanzhou, Gansu, China; ^3^ Department of Cardiology, Gansu Provincial Hospital, Lanzhou, Gansu, China

**Keywords:** TSH, SERCA2a, cardiomyocyte, PLN, PKA, Pathology Section

## Abstract

Elevated thyroid-stimulating hormone (TSH) levels often accompany impaired LV diastolic function and subtle systolic dysfunction in subclinical hypothyroidism (sHT). These cardiac dysfunctions are characterized by increases in mean aortic acceleration and pre-ejection/ejection time ratios. To explore the mechanism underlying these pathologies, we investigated the effects of TSH on sarcoplasmic reticulum calcium ATPase (SERCA2a) activity and expression in neonatal rat cardiomyocytes. TSH inhibited SERCA2a activity and expression by binding to TSH receptors in cardiomyocyte membranes and inhibiting the protein kinase A/phoshpolamban (PKA/PLN) signaling pathway. These results suggest that increases in serum TSH levels contribute to the development of cardiac diastolic and systolic dysfunction.

## INTRODUCTION

Hypothyroidism is characterized by elevated serum thyroid stimulating hormone (TSH) concentrations and reduced concentrations of free T4. Several studies show that hypothyroidism changes myocardial oxygen consumption, cardiac output, and cardiac contractility, resulting in decreased heart output along with a prolonged isovolumic relaxation phase. Some of these cardiovascular symptoms result from the impact of thyroid hormone on the cardiovascular system [1].

Interestingly, we found that the risk of these cardiovascular events is also increased in subclinical hypothyroidism (sHT), which is defined by elevated serum TSH with normal free thyroid hormone levels. In sHT, the most common cardiac abnormality is an impairment in LV systolic function, characterized by increases in mean aortic acceleration and pre-ejection/ejection time ratio both at rest and during exercise [2, 3]. Similarly, sHT is also associated with diastolic dysfunction, characterized by impaired early ventricular filling and slowed myocardial relaxation at rest that becomes more pronounced during exercise [4–7]. Conventional echocardiography and videodensitometric analysis confirmed that myocardial contractility is reduced and both the active and passive phases of diastole are dysregulated in sHT, as indicated by the following: (a) a lower cyclic variation index (CVI) in sHT indicated altered myocardial intrinsic contractility; (b) lower systolic strain and strain rate revealed an impairment of myocardial regional deformability; (c) an increase in peak A and decrease in peak E mitral flow velocity that resulted in a decline in left ventricular diastolic function [8].

Caraccio *et al*. described how long-term increases in serum TSH concentration contribute to the development of sHT [9]; TSH alone negatively impacted cardiac function, independent of HT. Moreover, exposure to slightly elevated TSH levels may, over time, contribute to systolic and diastolic abnormalities in sTH. These abnormalities often stem from changes in calcium homeostasis caused by altered expression or function of calcium transport or binding proteins. One such protein, cardiac sarcoplasmic reticulum calcium ATPase (SERCA2a), is a crucial for both cardiac contractility and the restoration of cytosolic calcium concentration during relaxation. The purpose of this study was to explore whether and how TSH regulates the expression of SERCA2a in neonatal rat cardiomyocytes. A better understanding of the cellular effects of TSH on the heart and cardiovascular system may contribute to the treatment of cardiac contractility and relaxation abnormalities associated with sHT.

## RESULTS

### Neonatal rat cardiomyocyte isolation

**Figure 1:**
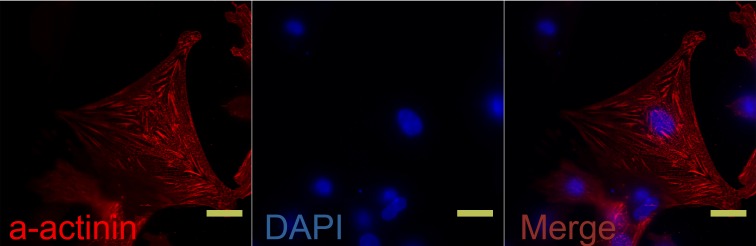
Isolated rat neonatal cardiomyocytes were stained with α-actinin antibody (FITC-conjugated) Nuclei were visualized with DAPI staining (blue). *N* = 6 per group. Scale bar, 20 μm.

Cardiomyocytes were separated from cardiac fibroblasts with a differential sticking wall method. Twenty hours after plating, automobile pulsing was visible in the adhered cardiomyocytes. To determine the purity of cardiomyocytes, we examined α-actinin antibody staining in the cells using immunofluorescence [11] (Figure 1).

### TSH receptor (TSHR) in NRCMs

**Figure 2:**
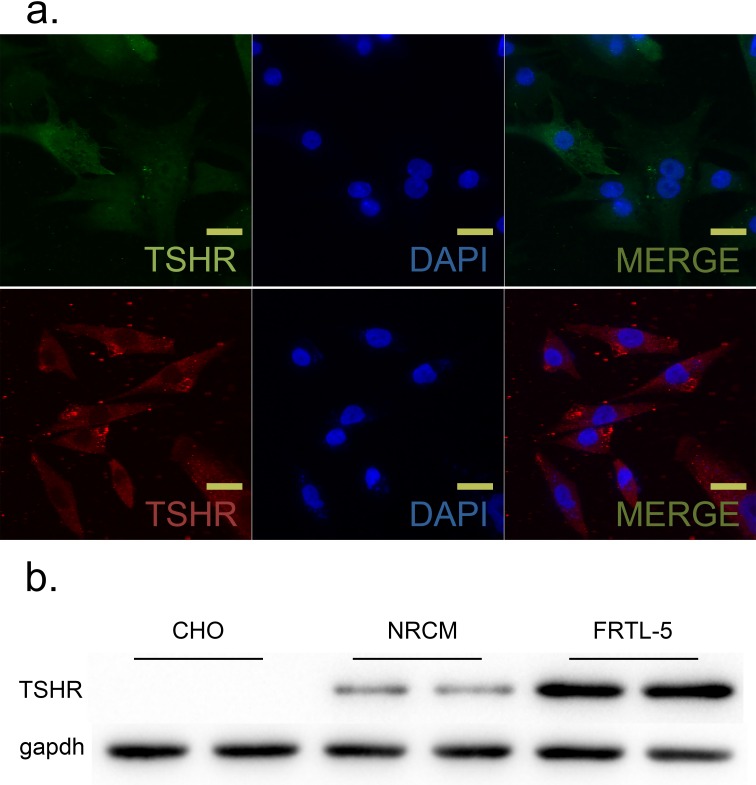
TSH receptor (TSHR) was expressed in neonatal rat ventricular myocytes (NRCM) and FRTL-5 cells GAPDH was used as an internal reference. **a.** TSHR was visualized using immunofluorescence in NRCM and TFRTL-5 cells. Upper, TSHR (cy3-conjugated) was localized at NRCM cell membranes. Lower, TSHR (FITC-conjugated) was localized at FRTL-5 cell membranes. Nuclei were visualized with DAPI staining (blue). *N* = 6 per group. Scale bar, 20 μm. **b.** TSHR protein levels in NRCMs were measured with western blots. Chinese Hamster Ovary (CHO) and FRTL-5 cells were used as negative and positive controls, respectively. *N* = 3 per group.

Although a distinct TSHR protein band was present in western blots from NRCM cells, the TSHR protein level was lower in NRCM cells than in positive control FRTL-5 cells; the TSHR band was not present in negative control CHO cells (Figure 2b). Immunofluorescent microscopy confirmed the presence of TSHR protein (red) in NRCM cell membranes (Figure 2a).

### TSH suppressed SERCA2a expression in NRCMs

**Figure 3:**
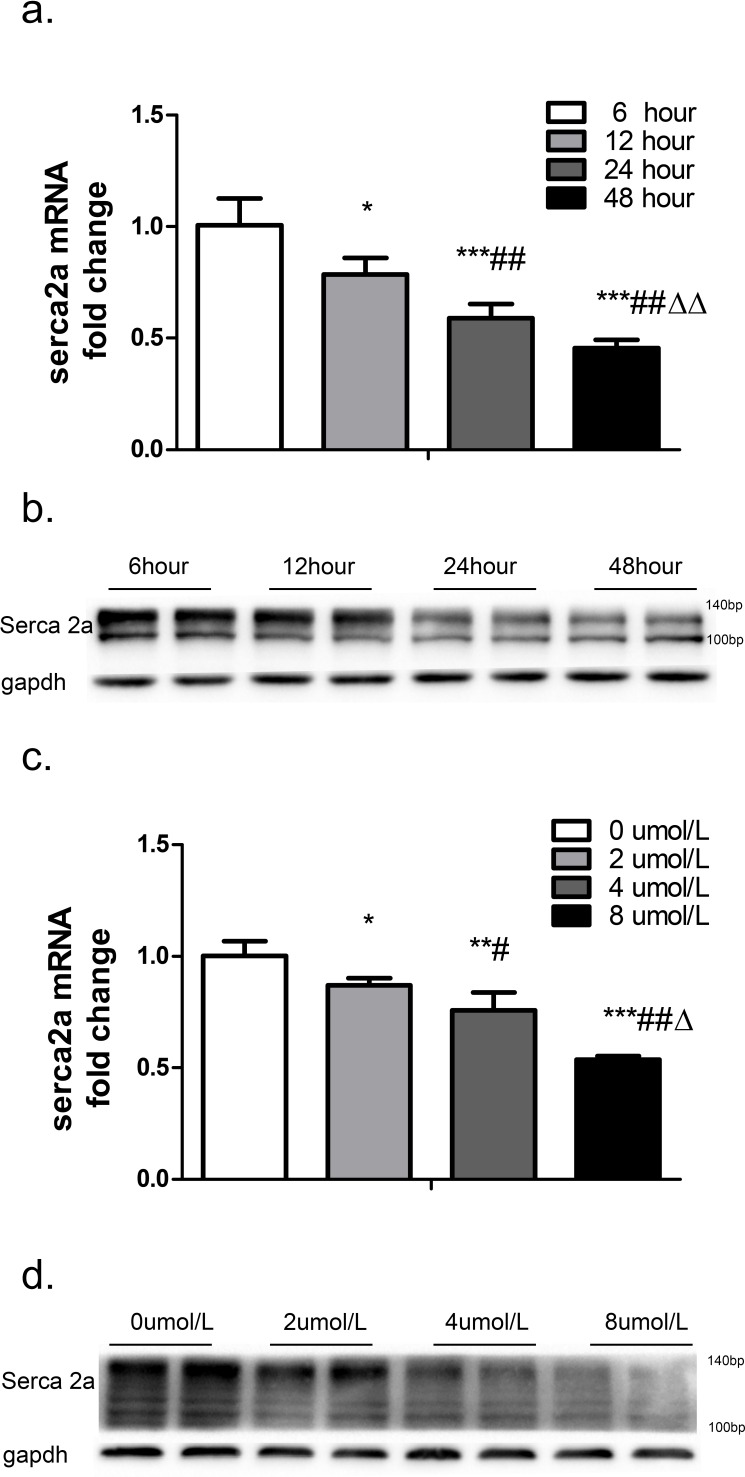
SERCA2a expression after treatment with different concentrations of TSH for different periods of time was measured in cardiomyocytes GAPDH was used as an internal reference. SERCA2a mRNA **a.** and protein **b.** levels were measured after treatment with 4 μM TSH for 6, 12, 24, or 48 hours. *p < 0.05, ***p < 0.001 *versus* control; *##p <* 0.01 *versus* 12-hour group; ΔΔ*p* < 0.01 *versus* 24-hour group. SERCA2a mRNA **c.** and protein (d) levels after treatment with 0, 2, 4, or 8 μM TSH for 48 hours. *p < 0.05 ,**p < 0.01, ***p < 0.001 *versus* 0 μM TSH. *#p <* 0.05, *##p <* 0.01 *versus* 2 μM group. Δ*p* < 0.05 *versus* 4 μM group. N = 6 per group for qPCRs and N = 3 per group for western blots. μM : μmol/L.

To explore the effect of TSH on ventricular SERCA2a expression *in vitro*, neonatal rat cardiomyocytes were treated with 4 μM TSH for different periods of time. Real-time PCR revealed that SERCA2a mRNA expression decreased by 22.01% in response to 12 hours (*p <* 0.05), 41.24% in response to 24 hours (*p* < 0.001), and 54.56% in response to 48 hours (*p* < 0.001) of TSH treatment compared to the 6-hour control treatment (Figure 3a). SERCA2a protein levels also decreased in a time-dependent manner after treatment (Figure 3b).

Cardiomyocytes were then treated with various concentrations of TSH for 48 hours. Real-time PCR showed that SERCA2a mRNA expression decreased by 13.09% in response to 2 μM (*p* < 0.05), 24.13% in response to 4 μM (*p* < 0.01), and 46.39% in response to 8 μM (*p* < 0.001) TSH compared to the control 0 μM treatment (Figure 3c). Western blot showed that SERCA2a protein levels also decreased in a dose-dependent manner after treatment (Figure 3d).

### TSH suppressed SERCA2a activity in NRCMs

SERCA2a activity decreased dose-dependently relative to maximal ATPase activity after 48 hours of treatment with 2 μM (65.1±6.6 nmol·mg^−1^·min^−1^) (*p* < 0.05), 4 μM (48.5±7.2 nmol·mg^−1^·min^−1^) (*p* < 0.01), or 8 μM (33.6±4.5 nmol·mg^−1^·min^−1^) (*p* < 0.01) TSH compared to control treatment (77.4±8.9 nmol·mg^−1^·min^−1^).

### TSH inhibited the expression of SERCA2a *via* the PKA/PLN pathway

**Figure 4:**
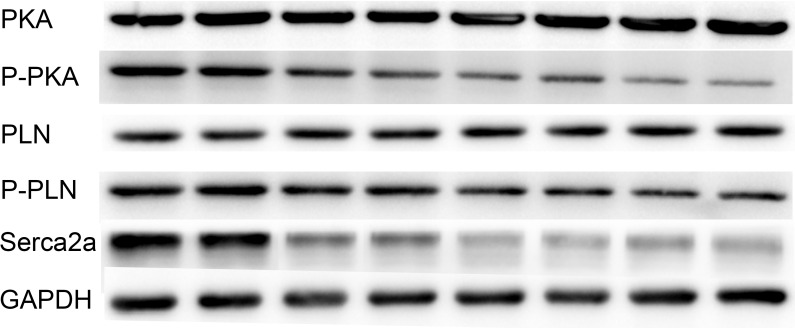
TSH inhibits SERCA2a expression by inhibiting the PKA/PLN pathway GAPDH was used as an internal reference. *N* = 3 per group. PKA: protein kinase A; PLN: phoshpolamban.

To explore the cell signaling pathway involved in the downregulation of SERCA2a in cardiomyocytes, we treated the cells with various concentrations (0, 2, 4, or 8 μM) of TSH for 48 hours and measured PKA, P-PKA, PLN, P-PLN, and SERCA2a protein levels using western blots. TSH dose-dependently decreased P-PKA, P-PLN, and SERCA2a protein levels, but not PKA or PLN levels (Figure 4).

**Figure 5:**
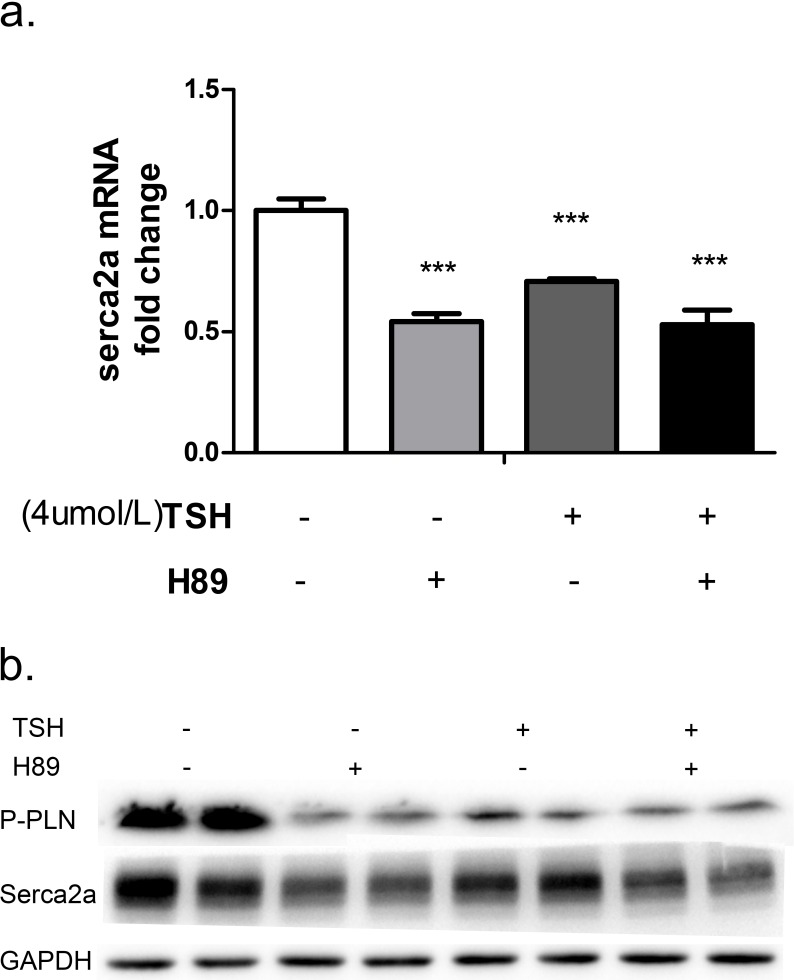
NRCMs were treated with 4 μM TSH and 20 μM H89 for 24 hours Changes in PKA/PLN pathway molecules and SERCA2a were measured with real-time PCR **a.** and western blot **b.** ***p < 0.001 *versus* control. GAPDH was used for normalization. *N* = 6 per group for qPCRs and *N* = 3 per group for western blots.

Similarly, treatment with a PKA inhibitor (H89) dramatically reduced P-PLN and SERCA2a mRNA (Figure 5a) and protein (Figure 5b) levels in NRCMs. To evaluate whether TSH suppressed SERCA2a by inhibiting Ser16 phosphorylation in PLN, we treated the cells with TSH and H89 simultaneously. This treatment decreased P-PLN and SERCA2a mRNA and protein levels as measured by PCR and western blot (Figure 5a, 5b). These results suggest that TSH decreases P-PLN and sesrca2a levels in NRCMs through a PKA-dependent pathway.

## DISCUSSION

TSH receptors (TSHRs) are primarily expressed in thyroid follicular cells, and their activation by TSH regulates the growth and functions of these cells. TSHRs are also present in extra-thyroidal cells, such as hepatocytes [12], lymphocytes [13], adipocytes [14], and retroocular fibroblasts [15]. Classic receptor binding studies demonstrated that TSHRs are also present in cardiac muscle [16, 17], and a recent study showed that TSHRs are expressed in H9C2 cells as well [18].

In this study, we extracted high-quality protein from NRCM cells and utilized optimized antibodies and western blots to investigate the presence of TSHRs and the mechanisms by which TSH regulates myocardial function. Western blot and immunofluorescence data indicated that TSHRs expressed in ventricular myocytes and provided important insights into pathophysiological roles of TSH in cardiac function.

In initial experiments, we used H9C2 cells to investigate TSH-induced cardiac damage. However, TSH did not affect H9C2 cell function. This may be due to the lack of automobile pulsing activity in H9C2 cells, which limited their use as a model for investigating pathological processes in the heart in this study.

The contractile function of cardiomyocytes is controlled by excitation-contraction (EC) coupling. During this process, calcium is removed from the cytosol; approximately 30% is transported out of the cell (primarily by the sodium-calcium exchanger [NCX] and plasma membrane calcium ATPase [PMCA]), while 70% is pumped back into the sarcoplasmic reticulum by the cardiac sarcoplasmic reticulum calcium ATPase (SERCA2a) [19]. SERCA2a is crucial for restoring cytosolic calcium concentrations during cardiac relaxation and contraction in cardiomyocytes [20, 21].

Perturbations of calcium homeostasis stemming from altered expression or function of calcium transport or binding proteins like SERCA2a are central to the development of systo-diastolic dysfunction [22, 23]. Additionally, the heart is the major target organ for thyroid disease. Recent clinical trials have shown an association between subtle systolic dysfunction and impaired LV diastolic function in sHT patients [24]. It is possible that these abnormalities in cardiac systo-diastolic function in sHT often result from TSH-induced alterations in SERCA2a expression.

Here, we observed that TSH dose- and time-dependently downregulated the activity and expression of SERCA2a mRNA and protein in cardiomyocytes. Long-term increases in TSH may therefore lead to subtle systolic dysfunction and damaged LV diastolic function in sHT patients by altering SERCA2a activity and cellular calcium transport. Moreover, recent evidence suggests that rising serum TSH levels might contribute to the development of sHT [9, 25]. Elevated TSH levels may contribute to cardiac dysfunction throughout the development and progression of hypothyroidism by initiating cardiomyocyte dysfunction in early-stage subclinical hypothyroidism and promoting subsequent pathological changes during later stages.

The small phosphoprotein phospholamban (PLN) modulates SERCA2a activity in cardiac muscle. Recent studies have revealed that sarcolipin (SLN), another small molecular weight protein, is also involved in SERCA2a regulation [26]. PLN expression is higher in the ventricle, which was the source of the cells used here [27], while SLN levels are higher in the atria [28–31]. PLN, a single-span membrane protein of only 52 amino acids, decreases the Ca^2+^ affinity of SERCA2a and attenuates contractile strength in the heart. PLN in turn is regulated by phosphorylation/dephosphorylation. In its dephosphorylated form, PLN binds SERCA2a and actively inhibits Ca^2+^-transport [32], and phosphorylation of PLN reverses this inhibition. PLN has two distinct phosphorylation sites: threonine 17, which is phosphorylated by Ca^2+^-calmodulin-dependent protein kinase (CaMKII) during β-adrenergic stimulation, and serine 16, which is phosphorylated by cAMP-dependent protein kinase (PKA) [33, 34]. Data from several studies show that reduced Ser16 phosphorylation correlates with reduced sensitivity of SERCA2a to high Ca^2+^ levels and leads to decreased myocardial contractility and relaxation abnormalities [35, 36]. Moreover, Ser16 phosphorylation is independent of CaMKII-dependent Thr17 phosphorylation, whereas the latter is dependent on preceding phosphorylation of Ser16 [15, 37]. Thus, Thr17 phosphorylation does not affect baseline PLN phosphorylation in normal tissues [38, 39]; we therefore focused on PKA-dependent phosphorylation of PLN at Ser16.

Here, we found that the PKA inhibitor H89 reduced PLN phosphorylation and SERCA2a expression, indicating that the regulation of P-PLN and SERCA2a is PKA-dependent. Co-treatment with TSH and H89 also inhibited the expression of P-PLN and SERCA2a in NRCMs. Furthermore, western blots confirmed that TSH dose-dependently decreased P-PKA, P-PLN, and SERCA2a protein levels. These data strongly suggest that TSH inhibits ventricular P-PLN and SERCA2a expression by inhibiting the PKA/PLN signaling pathway.

In summary, cardiomyocytes, which exhibit physiopathological responses nearly identical to those observed in the heart, were used as an *in vitro* model of cardiac function. We showed that TSHRs are expressed in cardiomyocytes and that TSH downregulated SERCA2a by inhibiting the PKA/PLN signaling pathway in these cells. Abnormally high serum TSH levels may therefore influence cardiovascular system function and may be useful as an independent risk factor for cardiovascular complications in hypothyroidism.

## MATERIALS AND METHODS

This experiment was approved by the ethical committees of the Nanjing Medical University, and all animal studies were completed in accordance with the recommendations of the Declaration of Helsinki and international standards regarding the use and care of laboratory animals.

### Neonatal rat cardiomyocyte isolation and culture

A principal culture of neonatal rat cardiomyocytes (NRCM) was obtained from the ventricles of 1-3 day old Wistar rats by enzymatic disassociation. Rats received heart surgery under 0.1 mL of 5% chloral hydrate anesthesia injected intraperitoneally. The atria and large vessels were removed from the detached hearts, which were then washed in 1x ADS solution (200 mL 10x ADS: 13.6g NaCl; 9.52 g HEPES; 0.276 g Na_2_HPO_4_; 1.2 g glucose; 0.8 g KCl; 0.0102 g MgSO_4_; 200 mL H_2_O, pH: 7.35-7.45). Cardiac cells were dispersed by a series of incubations at 37°C in 0.6 mg/mL pancreatin (Sigma-Aldrich Co., St. Louis, Missouri LLC. USA) and 0.5 mg/mL type II collagenase (Worthington, Lakewood, NJ, USA). The dispersed cells were plated differentially for 2 hours to reduce fibroblast contamination. Unattached cardiomyocytes were then seeded on 1% gelatin-coated plates in medium containing 10% horse serum, 5% fetal calf serum (Gibco Pasadena, CA, USA), and 5-Bromo-2′-Deoxyuridine (BRDU) (Sigma-Aldrich Co., St. Louis, Missouri, USA) (3 mg/mL) to further suppress fibroblasts. Twenty hours after plating, cells were transferred into serum-free medium to remove endogenous hormones and growth factors and then treated with or without various concentrations of bTSH (Sigma-Aldrich Co., St. Louis, Missouri, USA) for different periods of time. FRTL-5 rat thyroid cells were cultured at 37°C in a 5% CO_2_ humidified atmosphere in high-glucose DMEM supplemented with 10% fetal bovine serum (FBS), 100 U/ml penicillin, and 100 mg/mL streptomycin (Gibco Pasadena, CA, USA). These cells were utilized as the positive control in this study. The CHO cell line was cultured as previously described and served as a negative control.

### SERCA2a activity

The cardiomyocytes were lysed in 100 μL RIPA buffer (Beyotime Institute of Biotechnology) including a protease inhibitor cocktail (Sigma, St. Louis, MO, USA). The supernatant was then filtered by centrifuging at 12000 rpm for 15 min at 4°C. The protein was mixed with 10% sucrose buffer containing 0.5 mM MgCl, 400 mM KCl, 0.5 mM EGTA, 0.5 mM CaCl_2_, and 25 mM PIPES, pH 7.0. SERCA2a activity assays were based on a pyruvate/NADH coupled reaction. SERCA2a activity was calculated as Δabsorbance/(6.22×protein×time) in nmol ATP/(mg protein×min) [10].

### Quantitative RT-PCR

Total RNAs were isolated using RNAprep pure Cell/Bacteria Kit (TianGen, Beijing, China). To detect the expression of protein-coding genes by quantitative RT-PCR, 800 ng RNA samples were reverse-transcribed to cDNA with a Bio-Rad iScripTM cDNA Synthesis Kit (Bio-Rad, Hercules, CA, USA) according to the manufacturer's instructions in a reaction volume of 20 μL. The PCR protocol consisted of 3 minutes of denaturation at 95°C followed by 40 cycles of 95°C for 15 seconds, 60°C for 30 seconds, and 72°C for 30 seconds using the iQ™ SYBR^®^ Green supermixes (Bio-Rad, Hercules, CA, USA) in the 7900HT Fast Real-Time PCR System. Relative mRNA expression was calculated using the 2^−ΔΔCt^ method. The mRNA expression was normalized to GAPDH expression. The following primer sets were used for this experiment:

**Table 1:** Primer sequences used for real-time PCR

Gene	Primer sequence	Product (bp)	Gene bank
RatSERCA2a	5′-GGAGGCGTTGCTAAACACTC-3′ 5′-GAACCAGCCTTCGATATTGG-3′	201	AY-948198
RatGAPDH	5′-GGCACAGTCAAGGCTGAGAA-3′ 5-ATGGTGGTGAAGACGCCAGT-3′	143	NM-017008

### Western blotting

Cardiac cells were lysed by 12,000×g centrifugation for 15 min at 4°C in RIPA buffer (Beyotime Institute of Biotechnology) including a protease inhibitor cocktail (Sigma, St. Louis, MO, USA). Protein concentration was determined using a BCA Protein Assay Kit (Thermo Fisher Scientific, USA). Subsequently, samples were separated by SDS/PAGE and transferred to PVDF membranes which were incubated overnight at 4°C in 5% BSA with the following antibodies: Calcium Ion Regulation Antibody Sampler Kit (Cell Signaling Technology, Boston, Massachusetts, USA) (1:1,000); GAPDH (Abcam, Cambrige, UK) (1:1,000); and anti-TSHR (Santa Cruz Biotechnology, Dallas, TX, USA) (1:100). Samples were then washed three times with TBST buffer before adding secondary antibody. Using the ChemiDoc XRS Plus luminescent image analyzer (Bio-Rad, Hercules, CA, USA), specific protein bands were visualized using ECL Plus Western blot detection reagents (Bio-Rad). Densitometric analysis of band intensity was completed using Imagelab software (Bio-Rad, Hercules, CA, USA).

### Immunofluorescent microscopy

After the appropriate treatment, NRCMs were fixed with 4% paraformaldehyde for 20 min, then washed twice with PBS. Cells were permeabilized by incubation in 0.2% Triton X-100 in PBS for 20 min and incubated in 10% normal goat serum for 1 hour to block nonspecific binding. The samples were then incubated overnight at 4°C with the appropriate primary antibody, all of which were diluted with 10% normal goat serum: anti-actinin (Sigma, St. Louis, MO) (1:500), SERCA2a (Abcam, Cambrige, UK) (1:500), or anti-TSHR (1:50). Subsequently, the cells were incubated with secondary antibody at 37°C for 2 hours and the nuclei were stained with DAPI (Vector Laboratories, Burlingame, CA, USA). Cell images were captured using a confocal laser scanning microscope (CarlZeiss LSM710, Germany). All images were obtained using the same intensity and photo detector gain settings to allow quantitative comparison of immunoreactivity.

### Statistical analysis

One-way analysis of variance (ANOVA) and *t*-tests were conducted for statistical analysis using Graphpad Prism 5 software. Data are expressed as mean±SE. A *p* value of < 0.05 was considered significant.
